# 

**Published:** 1892-01-02

**Authors:** 


**Notices of Births, Deaths, and Marriages,** are inserted in this column at a charge of 2s. 6d. for an announcement not exceeding four lines.

### NOTICE TO CORRESPONDENTS.

All MS., Letters, Books for Review, and other matters intended for the Editor should be addressed THE EDITOR, THE LODGE, PORCHESTER SQUARE, LONDON, W.

The Editor cannot undertake to return rejected M.S., even when accompanied by stamped directed envelope.

**Notice to Advertisers and Subscribers.**

All Advertisements, Orders for Copies of THE HOSPITAL, and Business Communications should be addressed to The Publisher, *not to the Editor*, THE HOSPITAL, 140, Strand, W.C.

The Cost of Subscription, post free, is as follows:—Per week, 1½d.; per quarter, 1s. 8d.; per half-year, 3s. 3d.; per annum, 6s. 6d. Foreign:—Per annum, 8s. 8d.; payable, in all cases, in advance.

**"Burdett's Hospital Annual," 1891—1892.**

Third year of publication. 600 pp. Boards, gilt lettering. A most complete guide to British and Colonial Hospitals, Dispensaries. Nursing and Convalescent Institutions, and Asylums. Price 3s. 6d. Published by THE HOSPITAL (Limited), 140, Strand, W.C.

**NOTICE.—The Index for Vol. X. (ending September, 1891), is on sale at the Office of this Journal, price 2½d., post free.**

### General Charities.

[This Column is devoted to an account of work of charitable institutions other than Medical Charities. The Editor will be glad to receive reports or news of work at 140, Strand. *Philanthropy* should be plainly written on the envelope.]

The Lord Mayor has become a Vice-President of the Surgical Aid Society.

Mr. Algernon C. P. Coote has been appointed Secretary to the Orphan Working School, Haverstock Hill.

The sum of £5,000 has been generously given by an anonymous donor to the Special Fund which the National Society is raising for the assistance of poor and necessitous Church schools.

The fishermen of Grimsby are much gratified at the interest which the Prince of Wales has taken in the creation of the **ROYAL PROVIDENT FUND FOR SEA FISHERMEN**, and it is to be hoped that the inauguration of the scheme will be followed by a full measure of success.

The Royal National Lifeboat Institution has received among other sums recently, £4,600 from the Manchester Lifeboat Saturday Fund; £1,050 from Mrs. Thomas Simcox; £700 from Miss Curling; £250 from Mr. G. Buckstone Browne; and several contributions from "Lifeboat Sunday" collections following the example set by Manchester.

The London Needlework Guild has evidently not been idle during the past twelve months, for the Society has collected over 39,000 articles, which are now being distributed to the homes, refuges, missions, &c., which commend themselves to the institution as being in need of such assistance.

A sad record was presented to the last monthly meeting of the Committee of Management of **THE SHIPWRECKED MARINERS' SOCIETY**, which has helped during the past year 5,409 cases of distress caused by the shipwreck of 686 vessels. Many destitute widows and orphans of drowned sailors have been sought out by the Society, all of whom have received immediate succour, and many permanent relief. The need of such a Society has, during the past three months, been demonstrated in only too painful a manner.

The **SURGICAL AID SOCIETY** is to be congratulated upon the satisfactory condition of its finances. The report presented at the twenty-ninth annual meeting shows that the annual subscriptions are steadily increasing. As the Lord Mayor, who presided and appealed to the employers of labour to support the work of the Society, pointed out, the fact that 9,441 persons had been helped last year, and that over 101,000 persons have been assisted during the twenty-nine years in which the Society has been established, is sufficient to show how valuable the operations of the Society are.

The work which is carried on by the **SOCIETY OF ST. VINCENT DE PAUL** is in need of support. Facts which should commend themselves to those who have funds to contribute to deserving charities are that the members themselves contribute largely towards the maintenance of the Society (last year they contributed £1,744 towards an outlay of £8,200), and that the administration is carried on free of cost. Subscribers, therefore, will know that all the money they give will go to those whom they wish to benefit. The organisation exists for the purpose of relieving the numerous difficulties which beset working men and their families, and Lady Herbert will be grateful for any donations sent to her at Herbert House, Belgrave Square, S.W.

Appeals of varied kinds are constantly being made for help in the East End, and there is danger of a generous, but too credulous, public helping by its support to multiply agencies for the relief of distress to an undue extent. There must inevitably be a waste of strength as well as of money, when agencies aiming at similar ends are allowed to overlap one another. This, apparently, was in the mind of the Bishop of Bedford, who, while giving all due credit to Mr. Montagu Williams for the kindly motives which prompted him to appeal for blankets and old clothing for distribution among the poor in the East-end, wishes to remind the charitably disposed that a depôt already exists "at 180, Church Street, Stoke Newington, where all such things, old and new, are thankfully received." The Bishop will be glad to receive any charitable donations there, which will be dispensed through the clergy and others according to the needs of the different localities.

The Managers of the **LONDON ORPHAN ASYLUM, WATFORD**, earnestly appeal for help. During the present year 112 orphans have been admitted to the benefits of the charity, entailing a liability of £20,000. In addition to this responsibility, the Managers have been obliged to spend £900 towards perfecting the sanitary arrangements at the Asylum. A serious deficit at the end of the year appears, therefore, inevitable. Looking, however, to the large number of candidates seeking relief, it has been determined to admit 40 orphans at the ensuing election, in the confident hope that the required help will be forthcoming. It is to be hoped that the Committee are not thus acting rashly, such an extension of the work of the institution as is implied in this determination may still further increase the probable deficit at the end of the year, entailing further appeal to the public, already somewhat overburdened with such appeals. Five hundred and twelve orphans are now in the Asylum; 5,444 have been already maintained and educated. Contributions in aid will be very gratefully received by the Secretary, at the Office, 21, Great Saint Helen's, E.C.

### Scraps and Gleanings.

Yellow fever has appeared among the French troops in the French Soudan.

A new wing has been opened in the Mexborough Montagu Cottage Hospital.

Lord George Hamilton distributed the prizes to the girls of the Princess Helena College, Ealing.

A pretty entertainment was held in Victoria Hall, Weston-super-Mare, in aid of the hospital in that town.

The cyclists of Liverpool held an interesting spectacle in the shape of a masqued parade given in aid of the Stanley Hospital.

The proceeds of a sale of work in aid of the Tweed Bed Endowment Fund, arranged by the Countess of Home, amounted to £234.

The working classes of Wigan show a pleasing appreciation of their hospital by an increased subscription of £539 during the present year.

Last year the Christmas tree in aid of the Borough Hospital at Bootle was a great success. This year a similar entertainment is to be held.

The Duke of Westminster has accepted the office of president of the National Hospital for the Paralyzed and Epileptic (Albany Memorial), Queen Square, Bloomsbury.

It has finally been decided to erect a consumption hospital at Ballynafeigh, in Ireland. The fears of the public as to the danger of infection are confidently stated to be groundless.

A large fancy bazaar, in aid of the funds of the new European Hospital, was held at the Montgomery Hall, Lahore, on the 18th and 19th December, under the patronage of Lady Lyall.

The amount of the annual collections made in connection with the Hospital Saturday movement on behalf of the Wolverhampton General Hospital show, so far, a very gratifying result this year.

The minister of the East Free Church, Brechin, who has previously provided and endowed a bed in the Brechin Hospital, has intimated his intention to make a second gift of £20 annually for a like purpose.

The Duchess of Albany distributed the prizes to the successful scholars of Tiffins' Endowed School for Girls, Kingston-on-Thames, at the Surbiton Assembly Rooms, in the presence of a very large assembly of parents and visitors.

The Duke and Duchess of Portland attended the service at the parish church, Mansfield, on Hospital Day. The church was crowded to its utmost capacity. At the conclusion a collection was made for the benefit of the hospital, which realised £58.

The Earl of Pembroke has given £6,000 to build an addition to Baggot Street Hospital, Dublin, and £4,000 to form the nucleus of a fund to build a fishery school at Ringsend, Dublin, similar to that at Baltimore, presided over by Father Davis.

Mr. C. A. Palmer, Hon. District Secretary of the St. John's Ambulance Association, reported that during the last session 67 first-aid certificates had been obtained, while 12 of that number had been re-examined, of whom 11 had gained medallions.

Jane Glenney, an actor's wife, met her death recently through her head becoming submerged whilst taking her bath. It is almost to be wondered at that accidents of the kind do not more frequently happen. It would be well if a bell were fixed within easy reach of the hand in every bath-room.

Baron F. de Rothschild, M.P., presided at a meeting of the National Leprosy Fund. Sir Somers Vine reported the completion of the Memorial Cross to the late Father Damien, its free transmission to Honolulu, and the undertaking of the Hawaiian Government to erect it over the grave of the deceased priest.

A giant named Lepy, who resided at Lyons, was recently murdered. He was removed to the hospital, where, shortly before his death he bequeathed his body to the Faculty of Medicine at Lyons, as a slight return for the kindness which he had always met with from the people of that city.

The twelfth annual exhibition of home-made and other toys for distribution amongst the children in London hospitals, workhouses, workhouse schools, and infirmaries, through *Truth*, was held at the Polytechnic Institution, Regent Street, on Wednesday and Thursday last. There were some 23,000 to 24,000 toys on exhibition.

The London Homœopathic Hospital is at the present moment in debt to the extent of £700 (temporarily met by a loan), the in-patients of the hospital having increased progressively within the past five years from 500 to over 800 annually; the out-patients from 8,000 to 10,000 annually. An appeal for special donations or annual subscriptions is made.

Whilst the contributions at many of the London churches show a diminution in their contribution to the Hospital Sunday Fund, Dr. Forrest, vicar of St. Jude's, South Kensington (now Dean of Worcester) again headed the list of contributions with £1,300, which was the largest sum ever received by the Council from any one congregation.

A Reuter's telegram from Berlin states: In connection with the prevailing epidemic of influenza in Berlin a curious and somewhat disquieting fact has been reported. In the clinical wards of the Charité Hospital, under the superintendence of Professor Leyden, the disease has reappeared in the same rooms that were occupied by influenza patients in 1889.

The Lord Mayor presented the medallions and certificates awarded by the St. John Ambulance Association to members of the City of London Police Force, at the Mansion House yesterday afternoon. Lieutenant-Colonel Smith, Commissioner of the City Police, pointed out the necessity of a knowledge of ambulance work, especially to policemen. He had made it compulsory on every man on joining the force to undergo a course of this instruction.

An entertainment was given to the inmates and nursing staff of the British Home for Incurables, by the Rev. Prebendary Gordon Calthrop, last week. The Board of Management earnestly ask for contributions, however small, towards the fund for the general purposes of this charity, which distributes a pension of £20 per annum to nearly 300 incurable sufferers in all parts of the United Kingdom, besides providing for the inmates of the home at Clapham.

### THE STUDENT OF MEDICINE.

[All communications for this Supplement should be addressed to THE EDITOR of THE HOSPITAL, at 140, Strand, with the words, "Students' Column," written in the left hand top corner of the envelope.]

### NOTES FROM THE SCHOOLS.

### Edinburgh University.

A. Steele, G. S. Thorn, and H. Case, of the University R.F.C., were chosen to play for Edinburgh against Glasgow on December 5th.

The volunteer corps in connection with the University—the 2nd Battery Edinburgh City Artillery Volunteers, No. 2 Company Queen's Edinburgh Rifle Volunteer Brigade (Royal Scots), and the 2nd Division Volunteer Medical Staff Corps held a combined on December 4th, Surgeon-Captain D. Hepburn in the chair.

There has been a correspondence in "The Student" lately on the cost of spending a year at this University. An arts student says it can be done for £50. A common or garden medical student thinks that in medicine £110 is enough without extras. In medicine there is both a summer and winter session, in arts only a winter session, and fees in medicine are much higher than in arts.

The following office bearers were elected for 1891-92 at the Union annual elections on 4th December: President, Mr. George Porter; secretary, Mr. H. O. Izard.

The Union Dramatic Society's second festival was in every way a success. On the 9th and 11th December, Sir Charles Young's drama, "Jim the Penman," was performed. Mr. Lander Lindsay as Baron Hutfeld, Mr. J. Wilson Shiels as James Ralston, and Mr. J. G. Walker as Captain Redwood, were great successes. In the feminine parts Miss Alice Brickmann's professional training showed itself conspicuously.

On the 10th and 12th December "Dream Faces" and "The Snowball," were given. In "Dream Faces" Mr. J. Wilson Shiels and Miss Alice Brickmann were again to the fore.

In "The Snowball," Felix Featherstone and Uncle John were very successfully rendered by Mr. A. Stodart Walker and Mr. W. Lyall Wilson. Miss Alice Brickmann was a charming Mrs. Featherstone.

The University orchestra, under Mr. Raoul de Dreux Kunz's conductorship, performed the instrumental and other music. The incidental music was composed by Mr. Kunz.

### The London Dental Hospital.

A most successful smoking concert was given on Saturday night, December 12th, by the Hospital Athletic Club, in the Marble Hall of the Adelphi Restaurant. Mr. Woodhouse Braine was in the chair. The hall was well filled, and the performance appeared to be greatly appreciated by the students, who were present in large numbers. The programme was a very varied one for a smoking concert, and although one or two professionals gave their services, the greater part was entirely performed by the students of the Dental Hospital and their friends. A somewhat novel item was introduced into the evening's entertainment, in the way of some demonstrations of "Thought Transference," by Mr. Herschell, a student of the hospital, who was introduced to the audience by his brother, Dr. Herschell. Many of his experiments were certainly very clever, and by no means easy of ordinary explanation. Mr. Herschell first went through the usual tricks, such as, when blindfolded, telling a card drawn from a pack by his brother, without any contact. Out of several tries Mr. Herschell was only once wrong as to the card drawn. So also he was wonderfully correct in telling the dates of coins, such as pennies, when asked by any of the audience. Finally Mr. Herschell, also blindfolded, superintended a game of nap between two gentlemen, telling each in turn what card to play and who would win. Considerable talent was shown in the singing, and special mention must be made of Mr. Barnett's "Seize the Opportunity" and "She's the Boss, I'm the Slavey." So also Mr. H. Goodman sang "You're a Liarty" and "The Irish Jubilee" in great style. Two banjo duets were played by Mr. O. C. Sibley, of the Middlesex Hospital, and Mr. Ackland, the first, the "Black Brigade March," and the second, the well-known "Park Crescent March." Both of these pieces were admirably played, and showed great talent. Mr. Sibley we consider one of the best amateur banjoists we ever heard. The Musical Society gave two glees, "Oh, Who will O'er the Downs so Free" and "The Man of Harroch." Mr. Herschell also entertained the audience with several exceedingly well-rendered songs, including "Such a Nice Man," "Our 'Armonic Club," and "He Wasn't Used to Luxuries." Among the other performers mention

### The Hospital Advertiser.

### THE STUDENT OF MEDICINE—(*continued*).

must be made of a piano solo by Mr. Pascal Taylor, songs by Messrs. Webster, Hankey, Henley, Searle, Hope, and Hall.

### THE STUDENT'S BOOKSHELF.

**Modern Materia Medica for Pharmacists, Medical Men, and Students.***

At the present day, when so many new drugs have been introduced, a small manual of this kind must be welcome to all students. This book describes all of the recent preparations, most of them not having as yet found their way into the British Pharmacopœia. We are reminded in the introduction that many of the natural drugs are being superseded by artificial ones or those made in the chemist's laboratory. Among the very large number of compounds so produced, some have been found capable of replacing the natural alkaloid in many cases, while in others they seem to be even superior to it in therapeutic activity, reliability, or safety. In the small work before us, all the new alkaloids are separately described and arranged in alphabetical order, commencing with acetanilide and finishing with urethane. In each case the chemical formulæ, together with the synonymous terms of the preparation, are given. Then the method of preparation is described, together with the physical and chemical properties, and finally the medicinal uses. Some of the full chemical names of these new drugs are of great length, one, for instance, having twenty-nine letters in it. Many of the names, however, denote very clearly the action or use of the preparation such as antifebrin, antisepsin, disinfectol, diuretin, exalgin, hypnal, and many others.

A table of the average doses of new remedies is given both for single doses, and the amount that may be taken in the day, also a table of the dosage of antipyretics for children, and then a table of the solubility of new remedies both in water and also in spirit, together with some general remarks thereon; finally, a table is appended of the melting and boiling points of these drugs. The whole book is very clearly and explicitly written and well arranged, being also carefully indexed, and should fill a much-felt gap in works in this branch of medicine. We most cordially recommend it to both students and practitioners—to the former to get a general view of the nature and character of remedies which are coming daily more and more into general use, and to the latter, in that special knowledge of many of the preparations required in order to prescribe them satisfactorily, and this is supplied by many examples of good combinations or special methods of prescribing some of the more insoluble forms.

* Modern Materia Medica for Pharmacists, Medical Men, and Students, By H. Helbing, F.C.S. (Published by the *British and Colonial Druggist*. 21, New Bridge Street, London, E.C.)

### APPOINTMENTS.

At the City of London Hospital for Diseases of the Chest Mr. T. H. Arnold Chaplin, M.B., Cantab., M.R.C.P., has been appointed to the office of pathologist in succession to Dr. W. J. Hadley, Mr. E. C. Williams, M.B., Cantab., &c., has been appointed to the office of house physician for six months from 1st January to 30th June, 1892.

### SCHOLARSHIPS AND PRIZES.

#### Westminster Hospital.

Honours List, 1890-1.—Entrance Scholarships—Winter: A. T. White (£80), A. H. Evans, A. J. Smith, equal (£40), G. F. S. Genge (Epsom). Summer: L. B. Betts (£40). Treasurer's Prize, for first winter subjects (value 10 guineas), R. H. Norman. President's Prize, for second-year subjects (value 20 guineas), F. K. Wilson, H. M. Dowler, M. Farrant, *prox. accesserunt*. Bird Prize and Medal (value £14), W. H. A. Tebbs. Chadwick Prize (value 20 guineas), J. B. Byles. Clinical Medicine Prize (value £5), H. H, Mills. Clinical Surgery Prize (value £5), H. A. Stonham. Class prizes and certificates—Anatomy, senior class, H. M. Dowler, M. Farrant, F. K. Wilson; junior class, R. H. Norman. Physiology, senior class, F. K. Wilson, H. M. Dowler, M. Farrant, A. W. Austin; junior class, R. H. Norman, S. G. Tippett, G. F. S. Genge. Histology, senior class, F. K. Wilson, M. Farrant, H. M. Dowler. Chemistry, junior class, R. H Norman, A. J. Smith. Medicine, senior class, H. A. Stonham, H. H. Mills, equal (prize). Surgery, senior class, H. A. Stonham (prize). Summer Session, 1891.—Midwifery, S. A. Bull (prize). Forensic Medicine Toxicology, S. A. Bull (prize). Pathology, S. A. Bull (prize). Histology, R. H. Norman (prize). Practical Chemistry, R. H. Norman, A. J. Smith, equal (prize). Materia Medica, R. H. Norman (prize).

#### London School of Dental Surgery.

Prize Winners, 1890. — Saunders' Scholars', Mr. T. Coysh, Mr. A. C. Gask, Mr. W. May; Ashs' Prize (given by Messrs. Ash and Sons), Mr. E. R. Bull; second prize (given by school), Mr. W. May; certificates of honour, Mr. A. C. Gask. Class Prizes, Winter Session, 1889 90.—Mechanical Dentistry: First prize, Mr. T. Coysh, Mr. C. Schelling.

### THE STUDENT OF MEDICINE—(*continued*).

Mettallurgy : first Mr. A. C. Gask ; second, Mr. C. Schelling. Operative Dental Surgery : First, Mr. W. May ; second, Mr. E. H. L. Briault. Class Prizes, Summer Session, 1890.—Dental Surgery : First prize, Mr. T. Coysh, Mr. A. C. Gask. Dental Anatomy : First, Mr. W. May; second, Mr. T. Coysh. Students' Society Prize.—Mr. M. Woolf. Prize Winners, 1891.—Saunders' Scholar, Mr. R. M. Gracey ; Ashs' Prize (given by Messrs. Ash and Sons). Mr. E. Balding. Class Prizes, Winter Session, 1890-91.—Mechanical Dentistry : First prize, Mr. C. J. Allin ; second prize, Mr. E. Balding ; certificates, Mr. F. T. Ladmore and Mr. A. P. Spurr. Metallurgy : First prize, Mr. T. A. Coysh : second prize, Mr. R. N. Gracey. Prizes in Operative Dental Surgery.—First prize. Mr. E. Gardner ; second prize, Mr. T. A. Coysh ; certificate, Mr. E. Balding. Class Prizes, Summer Session, 1891.—Dental Surgery : First prize, Mr. R. N. Gracey ; second prize. Mr. A. P. Spurr ; certificates, Mr. S. H. Hayward, Mr. E. Balding, Mr. F. T. Trott, Mr. C. S. Reed, Mr. E. W. Harwood. Dental Anatomy : First prize, Mr. R. N. Gracey; second prize, Mr. S. H. Hayward ; certificates, Mr. E. W. Harwood, Mr. C. H. Watson, Mr. E. Balding, Mr. F. T. Ladmore, and Mr. B. A. Castellote. Students' Society Prize, Mr. A. C. Gask.

### St. Mary's Hospital.

1890, entrance scholarship in natural science of 100 guineas, R. W. Dodgson; 1890, entrance scholarship in natural science of 50 guineas, J. C. Smellie: 1890, exhibition scholarship in natural science of 25 guineas, S. P. James; 1890, exhibition scholarship in natural science of 25 guineas, J. E. P. Davies ; 1890, University exhibition scholarship of 25 guineas, J. E. Beggs ; 1890, University exhibition scholarship of 25 guineas, W. Broadbent ; 1890, Epsom scholarship of value of £105, no award. Prizes.—Summer Session, 1890—Second year : Midwifery, F. J. Poynton; medical jurisprudence, J. Broadbent and J. S. Collier, equal. First year : Materia medica, W. C. Wood; botany, no competition ; practical chemistry, R. H. A. Atkin and J. N. Hall, equal ; histology, A. Stanley. Winter Session, 1890-91, — Third year : Medicine, J. Broadbent, B.A. ; surgery, E. A. Nathan ; practical surgery, E. A. Nathan and H. R. Power, equal ; pathology, J. Broadbent, B.A., and E. A. Nathan, equal. Third and fourth years : prize in ophthalmology of £10 10s., H. L. Hatch ; ditto, W. T. Atwool; pathology scholarships, J. O. Summerhayes and A. Senior. Second year : Anatomy, C. B. Hall ; physiology, F. Chown. First year : Anatomy, A. W. Sanders ; physiology, R. W. Dodgson. Special prizes, 1890.—Students of first year : for general proficiency in anatomy, physiology, materia medica, and chemistry, A. E. Wilson ; students of second year : for general proficiency in anatomy, physiology, midwifery, and medical jurisprudence, J. S. Collier ; students of third year : for general proficiency in medicine, surgery, pathology, and operative surgery, V. Warren Low. Prosectors, A. E. Wilson, W. C. Wood. Meadows' Memorial Prize.—E. A. Nathan (prize), J. Broadbent, B.A. (*prox. acc.*), N. E. Norway.

### St. Thomas's Hospital.

Entrance Science Scholarships.—P. J. Dear ranks as first scholar, but had previously obtained a free admission, certificate of honour ; W. E. Dixon, scholarship, 125 guineas, and certificate of honour ; H. C. Crouch, scholarship, £60, and certificate of honour. First Year's Students : K. J. Previté Orton, the William Tite Scholarship, £27 10s., and certificate of honour ; G. G. Genge, college prize, £20, and certificate of honour ; W. H. J. Paterson, college prize, £10, and certificate of honour. Second Year's Students : S. W. F. Richardson, the Musgrove Scholarship, £38 10s., and certificate of honour ; A. E. Russell, college prize, £20, and certificate of honour, and L. J. Miskin, college prize, £10, and certificate of honour, equal. Third Year's Students : E. M. Hainworth, college prize, £20, and certificate of honour ; E. Smith, college prize, £15, and certificate of honour ; C. Planck, college prize, £10, with second tenure of the Peacock Scholarship, and certificate of honour. Practical Medicine.—C. Latter, the Mead Medal, founded by Mr. and Mrs. Newman Smith. Surgery and Surgical Anatomy.—A Banks, the Cheselden Medal, founded by the late Mr. George Vaughan ; J. H. Fisher, special mention and certificate of honour. For General Proficiency and Good Conduct.—J. H. Fisher, the Treasurer's Gold Medal.

### PASS LISTS.

### Royal College of Surgeons of England.

The following gentlemen passed the first professional examination for the fellowship in anatomy and physiology at a meeting of the Board of Examiners on the 16th inst : F. E. A. Colby, student of Cambridge University and St. Bartholomew's Hospital ; A. W. W. Lea, M.R.C.S., of Owens College, and Royal Infirmary, Manchester ; R. W. Doyne, M.R.C.S., of Bristol School of Medicine and St. George's Hospital ; F. J. Fielder, of King's College. (Twelve candidates were referred back to their professional studies for six months.)

Passed on the 17th inst. : J. S. Collier, student of St. Mary's Hospital ; H. W. Lyle and L. V. Cargill, M.R.C.S., of King's College ; F. C. Kempson, of Cambridge University and St. George's Hospital ; J. J. Grace, of Otago University and St. Bartholomew's Hospital ; P. MacLeod Yearsley, M.R.C.S., of Westminster Hospital. (Ten candidates were referred back to their professional studies for six months.)

Passed on the 18th inst. : S. H. Hughes, M.R.C.S., student of St. Bartholomew's Hospital ; W. P. Purvis, M.R.C.S., and G. J. Arnold, of St. Thomas's Hospital ; H. M. Jordan, M.R.C.S., and A. J. Sharp, of Guy's Hospital ; C. B. Turner, of University College ; F. R. Riley, M.R.C.S., of London Hospital. (Nine candidates were referred back to their professional studies for six months.)

Forty-eight candidates presented themselves for the examination, seventeen of whom passed, and thirty-one were referred.

